# Standardization of a protocol for shotgun proteomic analysis of saliva

**DOI:** 10.1590/1678-7757-2017-0561

**Published:** 2018-05-07

**Authors:** Talita Mendes da Silva VENTURA, Nathalia Regina RIBEIRO, Aline Salgado DIONIZIO, Isabela Tomazini SABINO, Marília Afonso Rabelo BUZALAF

**Affiliations:** 1 Universidade de São Paulo, Faculdade de Odontologia de Bauru, Departamento de Ciências Biológicas, Bauru, São Paulo, Brasil.

**Keywords:** Methods, Proteomics, Standardization, Saliva

## Abstract

Saliva contains numerous proteins and peptides, each of them carries a number of biological functions that are very important in maintaining the oral cavity health and also yields information about both local and systemic diseases. Currently, proteomic analysis is the basis for large-scale identification of these proteins and discovery of new biomarkers for distinct diseases.

**Objective:**

This study compared methodologies to extract salivary proteins for proteomic analysis.

**Material and Methods:**

Saliva samples were collected from 10 healthy volunteers. In the first test, the necessity for using an albumin and IgG depletion column was evaluated, employing pooled samples from the 10 volunteers. In the second test, the analysis of the pooled samples was compared with individual analysis of one sample. Salivary proteins were extracted and processed for analysis by LC-ESI-MS/MS.

**Results:**

In the first test, we identified only 35 proteins using the albumin and IgG depletion column, while we identified 248 proteins without using the column. In the second test, the pooled sample identified 212 proteins, such as carbonic anhydrase 6, cystatin isoforms, histatins 1 and 3, lysozyme C, mucin 7, protein S100A8 and S100A9, and statherin, while individual analysis identified 239 proteins, among which are carbonic anhydrase 6, cystatin isoforms, histatin 1 and 3, lactotransferrin, lyzozyme C, mucin 7, protein S100A8 and S100A9, serotransferrin, and statherin.

**Conclusions:**

The standardization of protocol for salivary proteomic analysis was satisfactory, since the identification detected typical salivary proteins, among others. The results indicate that using the column for depletion of albumin and IgG is not necessary and that performing individual analysis of saliva samples is possible.

## Introduction

Saliva is a biological fluid composed of more than 99% water and less than 1% protein, electrolytes and other low-molecular-weight components. It originates mainly from three pairs of major salivary glands (parotid, submandibular and sublingual glands), as well as from 300 to 400 minor salivary glands present in the oral cavity. Saliva plays a key role in lubrication, chewing, swallowing and digestion. It protects the oral tissues and also provides biomarkers for local and systemic diseases^17^. Therefore, saliva contains more than 2000 proteins and peptides that are involved in an infinity of different biological functions in the oral cavity^17^. Saliva still plays a large role in the formation of acquired pellicle, which begins only a few seconds after exposure of the enamel to saliva^5^.

Human saliva is a biological fluid with enormous diagnostic potential. Because saliva can be noninvasively collected, it provides an attractive alternative for blood, serum or plasma^13^.

In the human saliva were identified 1166 proteins, and high portions of these proteins were found in serum. Currently, progress in salivary diagnostics has demonstrated that these contents can be very informative for detection of oral and systematic diseases^20^.

Proteomics, a new field of research centered on identification, quantitation, and characterization of proteins and their interplay, is largely based on the robustness, sensitivity, speed, and throughput of mass spectrometric procedures^6^. Currently, mass spectrometry is the basic technology for large-scale identification of these salivary proteins, and proteomic analysis of saliva has distinct advantages over blood, especially for proteins of low abundance^17^ ^,^ ^18^. One of the main challenges in proteomic analysis is the fact that highly abundant proteins can impair the identification of low-abundance proteins, considering the equipment dynamic range. In the case of saliva, albumin and immunoglobulin G (IgG), they are very abundant, and some authors have recommended using columns for depletion of these proteins during the extraction procedure^7^ ^,^ ^8^. Saliva functions are not only restricted to process food for digestion, considering that it contains a large number of proteins, which play important roles in the regulation of the immune defense and endocrine system and in the maintenance of mucosal tissue and dental health^1^.

Saliva may contain locally expressed proteins and other substances called biomarkers, which can be used as diseases' indicators, be closely related to an individual's health condition and change greatly when diseases occur. In general, most studies view saliva wrongly as a homogeneous body fluid. It is also not stable, but constantly in change, and its composition is affected among other things by sampling methodology, environment, periodicity, oral hygiene, psychological status and general health^6^ ^,^ ^13^ ^,^ ^20^.

Considering the importance of saliva in the oral cavity homeostasis, as well as its great potential as a diagnostic fluid, the aim of this study was to standardize a protocol to extract salivary proteins for further proteomic analysis. In the first test, we evaluated the need for using an albumin and IgG column to deplete these proteins during protein extraction. In the second test, we compared analysis of samples pooled from 10 volunteers with samples from individual analysis.

## Material and methods

### Ethical aspects and human subjects

The protocol of this study was submitted and approved by the Ethics Committee in Research with Human Beings of the Bauru School of Dentistry - FOB/USP (CAAE No. 61484116.0.0000.5417). Ten participants with good general and oral health took part of this study, which was based on previous *in vivo* studies^18^. Inclusion criteria were: nonsmokers with good general and oral health, stimulated salivary flow >1 mL/min and unstimulated salivary flow >0.25 mL/min, salivary pH>6.0.

### Saliva collection

The volunteers were asked to rest for 15 min before collecting saliva, sitting upright. They were asked not to speak or eat before beginning to collect saliva. First, they rinsed their mouths with 5 mL of drinking deionized water, then they were asked to swallow saliva for 5 min. After this period, the volunteers spit out all the saliva accumulated in the mouth in a plastic tube immersed in ice for 10 min (unstimulated flow). The saliva samples were immediately centrifuged at 14,000 g for 15 min at 4°C to remove all debris, such as insoluble material, cell debris and food debris. The supernatant from each sample was collected and frozen at -80°C until analysis. These procedures were based on previous studies^6^ ^,^ ^18^.

### Preparation of the saliva samples

The experiments were performed into two phases. The first test was done to evaluate whether or not the albumin & IgG Depletion SpinTrap column (GE Healthcare^®^, Buckinghamshire, UK) should be used. The second test was performed after the results of the first to compare analysis of salivary samples pooled from all the 10 volunteers with analysis of an individual sample from one selected volunteer.

For the first test, 100 μl of saliva from each volunteer was taken and transferred to 10 new tubes. For the second test, 100 μl of each saliva sample was also taken and transferred to 10 new tubes to constitute the pool, while 1 ml of saliva was taken from only one of the volunteers (randomly selected) for individual analysis.

Proteins from the saliva samples were extracted using an equal volume of a solution containing 6 M urea, 2 thiourea in 50 mM NH_4_HCO_3_ pH 7.8. The samples were vortexed at 4°C for 10 min, sonicated for 5 min and centrifuged at 14,000 g at 4°C for 10 min. This step was repeated once more. For the first test (with or without the use of the albumin and IgG depletion column), we added 100 μl of the extraction solution to each Eppendorf tube. For the second test (pool X individual analysis), we added 100 µl of the extraction solution in each Eppendorf tube (for the samples that will be pooled later on), while for the individual sample, we added 1 ml of the extraction solution. In all the cases, an equal volume of saliva sample and extraction solution was used. For the pooled samples, we placed the content of the 10 tubes in one tube after the extraction procedure, constituting the pool for further analysis.

After extraction, for the first test, the pooled sample was loaded into the albumin & IgG depletion columns, according to the manufacturer´s instructions Albumin & IgG Depletion SpinTrap column (GE Healthcare^®^, Buckinghamshire, UK). We did not use this column in the second test.

The samples were then concentrated to 150 μl in Falcon Amicon tubes (Merck Millipore^®^, Tullagreen, County Cork, Ireland). After concentration, the samples were reduced with 5 mM dithiothreitol (DTT) for 40 min at 37°C, alkylated with 10 mM iodoacetamide (IAA) for 30 min in the dark. After this procedure, we added 100 μl of 50 mM NH_4_HCO_3_, and the samples were digested with 2% (w/w) trypsin (Promega^®^, Madison, USA) for 14 hours at 37°C. After this period, we added 10 µl of 5% formic acid to stop the trypsin reaction, then the samples were purified and desalted using the C18 Spin columns (Thermo Scientific^®^, Rockford, Illinois, USA) and we withdrew a 1 ul aliquot of each sample from the tests for protein quantification by the Bradford method (Bio-Rad^®^, Hercules, Califórnia, USA)^16^. We resuspended the samples in the solution containing 3% acetonitrile and 0.1% formic acid to be submitted to Nano Liquid Chromatography Electron Spray Ionization Tandem Mass Spectrometry - LC-ESI-MS/MS (Waters, Manchester, New Hampshire, UK).

### Shotgun label-free quantitative proteomic analysis

Peptides identification was performed on a nanoACQUITY UPLC-Xevo QTof MS system (Waters, Manchester, New Hampshire, UK). The nanoACQUITY UPLC was equipped with nanoACQUITY HSS T3, analytical reverse phase column (75 μm X 150 mm, 1.8 μm particle size (Waters, Manchester, New Hampshire, UK). The column was equilibrated with mobile phase A (0.1% formic acid in water). Then, the peptides were separated with a linear gradient of 7-85% mobile phase B (0.1% formic acid in ACN) for 70 min at a flow rate of 0.35 μL/min. The column temperature was maintained at 55°C. The Xevo G2 Q-TOF mass spectrometer was operated in positive nano-electrospray ion mode, and data were collected using the MSE method in elevated energy (19-45 V), which allows data acquisition of both precursor and fragment ions, in one injection. Source conditions used included capillary voltage, 2.5 kV; sample cone, 30 V; extraction cone, 5.0 V and source temperature, 80^0^C. Data acquisition occurred over 70 min, and the scan range was 50–2000 Da. The lockspray, used to ensure accuracy and reproducibility, was run with a [Glu1] fibrinopeptide solution (1 pmol/μL) at a flow rate of 1 μL/min, as a reference ion in positive mode at m/z 785.8427. ProteinLynx Global Server (PLGS) version 3.0 was used to process and search the LC-MSE continuum data. Proteins were identified with the embedded ion accounting algorithm in the software and a search of the Homo sapiens database (reviewed only, UniProtKB/Swiss-Prot) downloaded on September 2015 from UniProtKB (http://www.uniprot.org/). The use of human database excludes the identification of bacterial proteins that could be present in the saliva.

## Results

In the first test, when the albumin and IgG depletion column was used, the total amount of protein recovered from the pooled samples after extraction was 8 μg, while only 35 salivary proteins were identified. Among them are proteins typically found in saliva, such as alpha-amylase 1 and 2B, cystatin isoforms, hemoglobin isoforms and mucin 7, among others (Table 1). When the depletion column was not used, the amount of protein recovered was much higher (48.0 μg) and 248 proteins were identified, among them many typical components of saliva such as alpha-amylase 1 and 2B, many cystatin isoforms, carbonic anhydrase 6, lactotransferrin, lysozyme C, mucin 7, proline-rich protein 4, protein S100A9, serotransferrin, statherin, several hemoglobin isoforms, among others (Table 2).

**Table 1:** Salivary proteins identified when the albumin and IgG depletion column was used

Accession number	Protein name	score	Cover (%)
P04745	Alpha-amylase 1	7589.70	54.99
P19961	Alpha-amylase 2B	6833.20	47.75
P04280	Basic salivary proline-rich protein 1	488.14	43.88
P02812	Basic salivary proline-rich protein 2	3642.44	45.67
P49407	Beta-arrestin-1	158.66	9.09
P01036	Cystatin-S	1465.11	31.91
P09228	Cystatin-AS	516.59	24.11
P01037	Cystatin-SN	1378.19	21.28
Q9UGM3	Deleted in malignant brain tumors 1 protein	98.93	2.11
P14867	Gamma-aminobutyric acid receptor subunit alpha-1	92.53	7.46
G3V1N2	HCG1745306_ isoform CRA_a	456.20	22.73
P69905	Hemoglobin subunit alpha	1306.87	28.17
P68871	Hemoglobin subunit beta	1659.66	66.67
P02042	Hemoglobin subunit delta	497.84	25.17
A0A0G2JMB2	Ig alpha-2 chain C region (Fragment)	559.94	16.76
P01876	Immunoglobulin heavy constant alpha 1	912.82	30.59
P01877	Immunoglobulin heavy constant alpha 2	345.30	20.00
P01591	Immunoglobulin J chain	1363.63	36.48
P01834	Immunoglobulin kappa constant	333.71	51.40
P0CG04	Immunoglobulin lambda constant 1	136.40	14.15
P0DOY2	Immunoglobulin lambda constant 2	165.46	23.58
P0DOY3	Immunoglobulin lambda constant 3	153.74	23.58
P0CF74	Immunoglobulin lambda constant 6	136.40	14.15
B9A064	Immunoglobulin lambda-like polypeptide 5	136.40	7.01
P31025	Lipocalin-1	1181.01	26.70
Q8TAX7	Mucin-7	95.21	3.71
P04746	Pancreatic alpha-amylase	6723.99	41.49
P01833	Polymeric immunoglobulin receptor	305.15	15.58
P12273	Prolactin-inducible protein	1027.80	40.41
A0A0A0MT31	Proline-rich protein 4	8108.76	72.29
Q5VSP4	Putative lipocalin 1-like protein 1	958.48	6.79
P02810	Salivary acidic proline-rich phosphoprotein 1/2	8108.76	72.29
P02814	Submaxillary gland androgen-regulated protein 3B	2090.48	65.82
A0A087WZY1	Uncharacterized protein	7158.08	16.60
Q96DA0	Zymogen granule protein 16 homolog B	721.70	41.83

Standardization of a protocol for shotgun proteomic analysis of saliva

**Table 2:** Salivary proteins identified when the albumin and IgG depletion column was not used

Accession number	Protein name	score	Cover (%)
Q15118	[Pyruvate dehydrogenase (acetyl-transferring)] kinase isozyme 1_ mitochondrial	89.50	8.26
P31946	14-3-3 protein beta/alpha	166.37	3.25
P62258	14-3-3 protein epsilon	177.85	3.14
Q04917	14-3-3 protein eta	166.37	3.25
P61981	14-3-3 protein gamma	166.37	3.24
P31947	14-3-3 protein sigma	166.37	3.23
P27348	14-3-3 protein theta	195.23	12.65
P63104	14-3-3 protein zeta/delta	166.37	3.27
Q6ZVK8	8-oxo-dGDP phosphatase NUDT18	138.11	19.50
E5KP25	A/G-specific adenine DNA glycosylase	242.24	5.28
P68032	Actin_ alpha cardiac muscle 1	10751.18	40.05
P68133	Actin_ alpha skeletal muscle	10681.87	33.95
P62736	Actin_ aortic smooth muscle	10396.48	37.14
P60709	Actin_ cytoplasmic 1	18715.02	66.67
P63261	Actin_ cytoplasmic 2	18715.02	66.67
P63267	Actin_ gamma-enteric smooth muscle	10327.17	31.12
Q6P461	Acyl-coenzyme A synthetase ACSM6_ mitochondrial	399.16	13.33
Q9UIF7	Adenine DNA glycosylase	242.24	5.31
Q9Y6U3	Adseverin	51.66	5.17
C9JKR2	Albumin_ isoform CRA_k	25004.47	77.94
P02763	Alpha-1-acid glycoprotein 1	259.49	7.46
P01009	Alpha-1-antitrypsin	114.17	14.59
P01023	Alpha-2-macroglobulin	195.37	14.25
P04745	Alpha-amylase 1	125762.3	77.69
P19961	Alpha-amylase 2B	85518.55	67.91
Q69YU3	Ankyrin repeat domain-containing protein 34A	213.80	23.19
Q5T3N1	Annexin (Fragment)	419.03	34.31
P04083	Annexin A1	454.28	33.53
P03973	Antileukoproteinase	822.96	40.15
Q16671	Anti-Muellerian hormone type-2 receptor	646.30	18.32
P02647	Apolipoprotein A-I	436.68	32.58
B1APP8	ATP-dependent 6-phosphofructokinase_ platelet type	156.72	21.29
O14965	Aurora kinase A	187.17	8.93
P04280	Basic salivary proline-rich protein 1	13742.73	44.39
P02812	Basic salivary proline-rich protein 2	36329.24	69.23
Q6W2J9	BCL-6 corepressor	171.50	2.34
P61769	Beta-2-microglobulin	7681.87	54.62
Q562R1	Beta-actin-like protein 2	1631.58	17.02
Q96DR5	BPI fold-containing family A member 2	4054.46	40.56
Q8TDL5	BPI fold-containing family B member 1	238.42	27.27
Q8N4F0	BPI fold-containing family B member 2	4941.71	32.97
Q8N4G4	CA6 protein	236.85	4.47
P23280	Carbonic anhydrase 6	1927.33	43.83
P07339	Cathepsin D	153.05	17.96
H0YDT2	Cathepsin W (Fragment)	152.45	12.32
A0A087X2B6	Cell cycle and apoptosis regulator protein 2	186.22	13.60
O60308	Centrosomal protein of 104 kDa	36.50	3.35
O94986	Centrosomal protein of 152 kDa	24.18	5.03
O75153	Clustered mitochondria protein homolog	864.26	9.93
P35606	Coatomer subunit beta'	186.05	6.73
G3V1A4	Cofilin 1 (Non-muscle)_ isoform CRA_a	613.65	18.79
P23528	Cofilin-1	613.65	16.87
Q8TD31	Coiled-coil alpha-helical rod protein 1	47.65	2.43
Q9P0B6	Coiled-coil domain-containing protein 167	170.32	15.46
P01024	Complement C3	181.96	9.32
Q2VPA4	Complement component receptor 1-like protein	148.59	7.21
P04080	Cystatin-B	3144.06	55.10
P01034	Cystatin-C	1547.12	31.51
P28325	Cystatin-D	535.37	47.89
P01036	Cystatin-S	41046.83	73.76
P09228	Cystatin-SA	21107.61	53.90
P01037	Cystatin-SN	40764.24	68.09
P54108	Cysteine-rich secretory protein 3	371.45	26.94
Q9UGM3	Deleted in malignant brain tumors 1 protein	274.04	6.80
Q8IYB7	DIS3-like exonuclease 2	192.96	5.42
Q9NVU0	DNA-directed RNA polymerase III subunit RPC5	187.74	4.66
Q1HG43	Dual oxidase maturation factor 1	248.89	13.12
O95714	E3 ubiquitin-protein ligase HERC2	190.34	5.05
Q8NG27	E3 ubiquitin-protein ligase Praja-1	680.83	14.31
P43897	Elongation factor Ts_ mitochondrial	129.02	9.23
Q0PNE2	Elongator complex protein 6	63.64	13.53
V9HW75	Epididymis secretory protein Li 109	337.33	22.86
P02675	Fibrinogen beta chain	420.77	40.73
P02679	Fibrinogen gamma chain	453.82	22.52
Q0PRL4	Forkhead box P2 variant 3	142.49	10.19
Q8N6B5	Forkhead box P2_ isoform CRA_d (Fragment)	142.49	11.84
O15409	Forkhead box protein P2	199.65	12.45
O95872	G patch domain and ankyrin repeat-containing protein 1	268.32	17.70
P19526	Galactoside 2-alpha-L-fucosyltransferase 1	174.70	13.42
P48058	Glutamate receptor 4	50.22	2.55
P04406	Glyceraldehyde-3-phosphate dehydrogenase	190.90	16.72
P00738	Haptoglobin	349.21	24.88
G3V1N2	HCG1745306_ isoform CRA_a	22783.57	58.18
P69905	Hemoglobin subunit alpha	27452.86	59.15
P68871	Hemoglobin subunit beta	49667.26	95.24
P02042	Hemoglobin subunit delta	9498.60	33.33
P02100	Hemoglobin subunit epsilon	1940.46	6.80
P69891	Hemoglobin subunit gamma-1	1940.46	6.80
P69892	Hemoglobin subunit gamma-2	1940.46	6.80
P02790	Hemopexin	460.96	22.51
P15515	Histatin-1	32092.25	36.84
P15516	Histatin-3	7558.25	13.73
P57058	Hormonally up-regulated neu tumor-associated kinase	218.10	3.50
Q9BS19	HPX protein	352.10	21.65
A0A0G2JMB2	Ig alpha-2 chain C region (Fragment)	22147.53	68.24
A0A0A0MS07	Ig gamma-1 chain C region (Fragment)	1490.66	45.76
A0A087WYJ9	Ig mu chain C region	2129.91	40.71
P04220	Ig mu heavy chain disease protein	1800.88	31.97
P01876	Immunoglobulin heavy constant alpha 1	25196.43	61.19
P01877	Immunoglobulin heavy constant alpha 2	18459.82	64.12
P01857	Immunoglobulin heavy constant gamma 1	3671.28	50.91
P01859	Immunoglobulin heavy constant gamma 2	729.35	38.34
P01860	Immunoglobulin heavy constant gamma 3	487.81	24.93
P01861	Immunoglobulin heavy constant gamma 4	599.47	20.18
P01871	Immunoglobulin heavy constant mu	2171.72	47.68
A0A075B7F0	Immunoglobulin heavy variable 3/OR16-10 (non-functional) (Fragment)	378.41	9.48
S4R460	Immunoglobulin heavy variable 3/OR16-9 (non-functional)	5403.28	31.25
P01762	Immunoglobulin heavy variable 3-11	378.41	9.40
P01766	Immunoglobulin heavy variable 3-13	378.41	9.48
A0A0C4DH32	Immunoglobulin heavy variable 3-20 (Fragment)	378.41	9.40
A0A0B4J1V1	Immunoglobulin heavy variable 3-21	378.41	9.40
A0A0B4J1X8	Immunoglobulin heavy variable 3-43	378.41	9.32
P01763	Immunoglobulin heavy variable 3-48	378.41	9.40
P01780	Immunoglobulin heavy variable 3-7	401.30	17.09
P01782	Immunoglobulin heavy variable 3-9	378.41	9.32
P01591	Immunoglobulin J chain	18415.28	42.14
P01834	Immunoglobulin kappa constant	16816.83	85.98
P0CG04	Immunoglobulin lambda constant 1	9338.45	77.36
P0DOY2	Immunoglobulin lambda constant 2	13921.14	77.36
P0DOY3	Immunoglobulin lambda constant 3	13921.14	77.36
P0CF74	Immunoglobulin lambda constant 6	13267.04	50.94
A0M8Q6	Immunoglobulin lambda constant 7	10499.89	36.79
B9A064	Immunoglobulin lambda-like polypeptide 5	9338.45	38.32
P08069	Insulin-like growth factor 1 receptor	32.75	5.63
P06870	Kallikrein-1	227.71	10.31
Q9Y5K2	Kallikrein-4	304.56	17.72
P13645	Keratin_ type I cytoskeletal 10	297.80	2.05
Q99456	Keratin_ type I cytoskeletal 12	421.18	14.17
P13646	Keratin_ type I cytoskeletal 13	4810.33	46.94
P02533	Keratin_ type I cytoskeletal 14	158.42	4.24
P19012	Keratin_ type I cytoskeletal 15	1164.86	14.25
P08779	Keratin_ type I cytoskeletal 16	158.42	4.23
Q04695	Keratin_ type I cytoskeletal 17	143.47	2.08
P08727	Keratin_ type I cytoskeletal 19	529.84	6.75
P35908	Keratin_ type II cytoskeletal 2 epidermal	300.25	22.07
Q01546	Keratin_ type II cytoskeletal 2 oral	165.14	12.07
P19013	Keratin_ type II cytoskeletal 4	876.71	42.13
P13647	Keratin_ type II cytoskeletal 5	489.99	7.97
P02538	Keratin_ type II cytoskeletal 6A	794.78	31.56
P04259	Keratin_ type II cytoskeletal 6B	765.88	28.01
P48668	Keratin_ type II cytoskeletal 6C	765.88	28.01
O95678	Keratin_ type II cytoskeletal 75	190.38	3.81
Q5XKE5	Keratin_ type II cytoskeletal 79	190.38	3.93
O14777	Kinetochore protein NDC80 homolog	410.89	9.03
P22079	Lactoperoxidase	1724.32	34.13
P02788	Lactotransferrin	382.65	32.11
Q9C099	Leucine-rich repeat and coiled-coil domain-containing protein 1	270.77	9.98
Q9NPC1	Leukotriene B4 receptor 2	209.15	4.37
P31025	Lipocalin-1	19334.38	57.95
P28330	Long-chain specific acyl-CoA dehydrogenase_ mitochondrial	137.44	9.07
Q8IYD9	Lung adenoma susceptibility protein 2	141.09	9.14
P61626	Lysozyme C	10190.75	70.27
Q14680	Maternal embryonic leucine zipper kinase	208.24	8.14
P42679	Megakaryocyte-associated tyrosine-protein kinase	156.39	10.85
P01033	Metalloproteinase inhibitor 1	858.61	44.44
Q2QL34	Mpv17-like protein	240.73	11.73
Q8TAX7	Mucin-7	11686.20	15.65
Q8NCY6	Myb/SANT-like DNA-binding domain-containing protein 4	176.81	11.30
P24158	Myeloblastin	175.85	4.69
Q8NCE2	Myotubularin-related protein 14	342.16	19.38
Q9NYA4	Myotubularin-related protein 4	234.57	15.82
F8WCT3	NEDD8-conjugating enzyme UBE2F	167.98	37.18
P59665	Neutrophil defensin 1	1037.46	17.02
P59666	Neutrophil defensin 3	1037.46	17.02
O00221	NF-kappa-B inhibitor epsilon	176.16	6.80
Q2L696	Nucb2 splice variant	337.33	24.62
Q14980	Nuclear mitotic apparatus protein 1	278.08	4.96
Q9Y618	Nuclear receptor corepressor 2	44.62	3.33
A0A087WSV8	Nucleobindin 2_ isoform CRA_b	337.33	22.86
P80303	Nucleobindin-2	337.33	22.86
O75414	Nucleoside diphosphate kinase 6	140.72	14.52
C9JQB1	Nucleoside diphosphate kinase	140.72	19.15
Q9GZK3	Olfactory receptor 2B2	166.49	19.33
Q5SZR7	Ornithine decarboxylase antizyme 3	300.95	18.55
Q7RTY7	Ovochymase-1	190.59	10.05
P04746	Pancreatic alpha-amylase	79860.79	59.10
P13796	Plastin-2	364.90	18.02
P13797	Plastin-3	259.13	4.29
P01833	Polymeric immunoglobulin receptor	10715.77	41.62
Q6S8J3	POTE ankyrin domain family member E	7556.27	11.07
A5A3E0	POTE ankyrin domain family member F	7557.11	13.67
P0CG38	POTE ankyrin domain family member I	6915.24	6.79
P0CG39	POTE ankyrin domain family member J	2868.60	5.97
P51531	Probable global transcription activator SNF2L2	158.85	2.01
Q53EL6	Programmed cell death protein 4	138.40	8.74
P12273	Prolactin-inducible protein	31682.10	76.71
Q16378	Proline-rich protein 4	312.60	21.64
H0Y4B9	Propionyl-CoA carboxylase alpha chain_ mitochondrial (Fragment)	231.31	20.90
P07602	Prosaposin	205.84	9.35
D6RDZ2	Protein FAM193B (Fragment)	266.86	35.56
Q14320	Protein FAM50A	176.55	10.62
Q5VT40	Protein FAM78B	141.80	10.73
Q8N7I0	Protein GVQW1	164.91	17.95
Q6P5S2	Protein LEG1 homolog	1162.24	29.09
Q8ND56	Protein LSM14 homolog A	270.50	9.50
Q8WYL5	Protein phosphatase Slingshot homolog 1	322.72	3.91
Q5THK1	Protein PRR14L	367.74	10.13
P06702	Protein S100-A9	571.65	39.47
Q96EA4	Protein Spindly	138.75	2.64
Q58EX7	Puratrophin-1	166.93	2.60
Q9BYX7	Putative beta-actin-like protein 3	1002.92	10.67
Q5VSP4	Putative lipocalin 1-like protein 1	3906.17	11.11
A8K554	Putative protein ZNF815	163.67	26.15
Q96GD0	Pyridoxal phosphate phosphatase	92.62	11.15
H3BR70	Pyruvate kinase	336.60	18.03
P14618	Pyruvate kinase PKM	336.60	12.43
Q15276	Rab GTPase-binding effector protein 1	349.01	8.24
H3BPI9	Receptor protein serine/threonine kinase (Fragment)	641.71	47.67
P02810	Salivary acidic proline-rich phosphoprotein 1/2	40463.03	26.51
Q14674	Separin	32.80	4.39
Q9BZL6	Serine/threonine-protein kinase D2	165.21	3.87
B4DTS2	Serine/threonine-protein kinase	165.21	3.83
J3QLP4	Serine/threonine-protein kinase RIO3 (Fragment)	335.03	50.56
G3V5U8	Serine/threonine-protein phosphatase 2A regulatory subunit B'' subunit gamma	157.81	24.53
P02787	Serotransferrin	5631.55	44.41
P02768	Serum albumin	65771.62	81.28
P40763	Signal transducer and activator of transcription 3	43.20	6.10
Q9UBC9	Small proline-rich protein 3	424.01	65.09
A1L4H1	Soluble scavenger receptor cysteine-rich domain-containing protein SSC5D	62.36	2.67
P02808	Statherin	52769.28	53.23
P02814	Submaxillary gland androgen-regulated protein 3B	52053.05	65.82
Q9UMS6	Synaptopodin-2	184.00	1.83
G5E9B5	TCF3 (E2A) fusion partner (In childhood Leukemia)_ isoform CRA_b	165.61	19.67
Q8WW35	Tctex1 domain-containing protein 2	188.69	14.08
Q7Z6L1	Tectonin beta-propeller repeat-containing protein 1	350.11	12.62
Q9UKR8	Tetraspanin-16	313.97	27.35
P20061	Transcobalamin-1	230.38	20.32
A6H8Y1	Transcription factor TFIIIB component B'' homolog	167.29	2.82
O95359	Transforming acidic coiled-coil-containing protein 2	372.27	6.41
P55072	Transitional endoplasmic reticulum ATPase	236.03	10.92
P29401	Transketolase	133.40	13.80
Q8NDV7	Trinucleotide repeat-containing gene 6A protein	180.44	3.98
K7EQY5	Tyrosine-protein kinase	156.39	10.87
Q86TW2	Uncharacterized aarF domain-containing protein kinase 1	174.76	10.57
H3BMD7	Uncharacterized protein (Fragment)	240.73	19.49
A0A087WZK3	Uncharacterized protein (Fragment)	469.24	43.09
A0A087WZY1	Uncharacterized protein	40463.03	16.60
A0A087WUV0	Uncharacterized protein	464.85	8.62
E7ESA3	Uncharacterized protein	188.69	18.87
Q9HB07	UPF0160 protein MYG1_ mitochondrial	435.46	12.23
Q9NY84	Vascular non-inflammatory molecule 3	540.71	10.58
Q14508	WAP four-disulfide core domain protein 2	1637.99	33.87
E9PDB0	WD repeat-containing protein 49	424.40	5.02
Q86UP3	Zinc finger homeobox protein 4	205.12	3.06
Q5FWF6	Zinc finger protein 789	138.52	9.41
Q17R98	Zinc finger protein 827	296.41	2.87
P25311	Zinc-alpha-2-glycoprotein	5026.17	55.03
Q96DA0	Zymogen granule protein 16 homolog B	47333.93	56.73

In the second test, for comparison of analysis of pooled *versus* individual sample, the depletion column was not used. For the pooled sample, the amount of protein recovered after extraction was 54.02 µg, which allowed the identification of 212 proteins, including alpha-amylase 1 and 2B, carbonic anhydrase 6, cystatin isoforms (B, C, D, S, SA, SN), histatin 1 and 3, lysozyme C, mucin 7, protein S100A8 and S100A9, statherin, several hemoglobin isoforms, among others (Table 3). In the analysis of the individual sample, 25.13 μg of total protein were obtained and 239 proteins were identified, among which are alpha-amylase 1 and 2B, alpha-enolase, carbonic anhydrase 6, many cystatin isoforms (B, C-D, S, SA, SN), histatin 1 and 3, Ig alpha-2 chain C region, Ig a chain C region, lactotransferrin, lysozyme C, mucin 7, protein S1008 and S100A9, serotransferrin, statherin, among other proteins (Table 4).

**Table 3:** Proteins of the saliva identified in the pool analysis

Accession number	Protein name	score	Cover(%)
P16885	1-phosphatidylinositol 4_5-bisphosphate phosphodiesterase gamma-2	314.78	4.51
P68032	Actin_ alpha cardiac muscle 1	6085.31	31.30
P68133	Actin_ alpha skeletal muscle	6085.31	31.30
P62736	Actin_ aortic smooth muscle	4676.94	28.38
P60709	Actin_ cytoplasmic 1	17496	67.20
P63261	Actin_ cytoplasmic 2	17496	67.20
P63267	Actin_ gamma-enteric smooth muscle	4676.94	28.46
Q01518	Adenylyl cyclase-associated protein 1	440.27	26.11
C9JKR2	Albumin_ isoform CRA_k	26466.72	74.82
P01009	Alpha-1-antitrypsin	2252.60	22.97
P01023	Alpha-2-macroglobulin	665.70	22.86
P04745	Alpha-amylase 1	153591.90	78.86
P19961	Alpha-amylase 2B	110753.50	58.51
P06733	Alpha-enolase	1637.76	33.87
Q01484	Ankyrin-2	52.62	2.75
P03973	Antileukoproteinase	701.53	28.03
P63010	AP-2 complex subunit beta	338.39	2.35
P02647	Apolipoprotein A-I	612.31	39.70
P02652	Apolipoprotein A-II	886.78	69.00
Q5FYB0	Arylsulfatase J	389.18	10.35
Q8IYB8	ATP-dependent RNA helicase SUPV3L1_ mitochondrial	235.17	6.23
P04280	Basic salivary proline-rich protein 1	3925.20	58.67
P02812	Basic salivary proline-rich protein 2	73554.97	69.47
P61769	Beta-2-microglobulin	3725.17	48.74
Q562R1	Beta-actin-like protein 2	1532.83	13.30
P13929	Beta-enolase	264.78	13.36
Q96DR5	BPI fold-containing family A member 2	4561.18	58.23
Q8N4F0	BPI fold-containing family B member 2	6508.75	30.79
A0A087WXK1	BRCA1-A complex subunit Abraxas (Fragment)	332.77	16.93
Q8N4G4	CA6 protein	419.28	4.47
O75638	Cancer/testis antigen 2	716.39	19.05
P23280	Carbonic anhydrase 6	15792.21	62.01
P00450	Ceruloplasmin	71.04	8.45
E9PM92	Chromosome 11 open reading frame 58	258.69	15.29
P01024	Complement C3	833.42	21.17
P51160	Cone cGMP-specific 3'_5'-cyclic phosphodiesterase subunit alpha'	232.14	11.07
H3BRY3	Coronin	502.10	22.11
P31146	Coronin-1A	502.10	24.95
Q92772	Cyclin-dependent kinase-like 2	457.97	11.97
P04080	Cystatin-B	2288.27	45.92
P01034	Cystatin-C	3131.85	51.37
P28325	Cystatin-D	3348.32	61.97
P01036	Cystatin-S	34860.66	73.76
P09228	Cystatin-SA	24277.69	67.38
P01037	Cystatin-SN	23133.23	70.21
P54108	Cysteine-rich secretory protein 3	284.38	21.63
P32320	Cytidine deaminase	1245.08	66.44
Q9UGM3	Deleted in malignant brain tumors 1 protein	306.82	4.97
Q13609	Deoxyribonuclease gamma	411.37	15.74
A0A0A0MT68	Deoxyribonuclease	411.37	16.67
P27487	Dipeptidyl peptidase 4	73.31	4.83
O60216	Double-strand-break repair protein rad21 homolog	322.95	19.02
R4GN68	Dual-specificity mitogen-activated protein kinase kinase 4	780.16	97.56
V9HW75	Epididymis secretory protein Li 109	954.67	25.48
B1AK53	Espin	277.28	4.80
Q01469	Fatty acid-binding protein_ epidermal	475.76	30.37
Q8NCQ5	F-box only protein 15	465.73	3.73
P02679	Fibrinogen gamma chain	372.17	21.63
Q08380	Galectin-3-binding protein	237.96	18.97
P06744	Glucose-6-phosphate isomerase	222.14	22.04
E7ETY7	Glutathione peroxidase	341.42	22.78
P09211	Glutathione S-transferase P	519.29	25.71
P04406	Glyceraldehyde-3-phosphate dehydrogenase	407.39	11.64
Q8IWJ2	GRIP and coiled-coil domain-containing protein 2	718.24	4.81
P00738	Haptoglobin	960.32	41.87
G3V1N2	HCG1745306_ isoform CRA_a	11936.33	57.27
P69905	Hemoglobin subunit alpha	13598.42	54.93
P68871	Hemoglobin subunit beta	18402.54	89.80
P02042	Hemoglobin subunit delta	5838.89	63.95
P02100	Hemoglobin subunit epsilon	3895.00	6.80
P69891	Hemoglobin subunit gamma-1	3895.00	6.80
P69892	Hemoglobin subunit gamma-2	3895.00	6.80
P15515	Histatin-1	16204.54	36.84
P15516	Histatin-3	2631.50	13.73
Q16695	Histone H3.1t	524.06	23.53
Q05469	Hormone-sensitive lipase	43.68	5.30
Q4G0P3	Hydrocephalus-inducing protein homolog	15.21	1.93
A0A0G2JMB2	Ig alpha-2 chain C region (Fragment)	43004.29	79.12
A0A0A0MS07	Ig gamma-1 chain C region (Fragment)	2528.80	42.37
A0A087WYJ9	Ig mu chain C region	4012.85	48.67
P04220	Ig mu heavy chain disease protein	3190.64	37.85
P01876	Immunoglobulin heavy constant alpha 1	38140.46	73.65
P01877	Immunoglobulin heavy constant alpha 2	32255.84	65.29
P01857	Immunoglobulin heavy constant gamma 1	4336.06	47.88
P01859	Immunoglobulin heavy constant gamma 2	1181.17	37.42
P01860	Immunoglobulin heavy constant gamma 3	1276.14	14.59
P01861	Immunoglobulin heavy constant gamma 4	1489.84	38.23
P01871	Immunoglobulin heavy constant mu	4017.99	50.33
A0A075B7F0	Immunoglobulin heavy variable 3/OR16-10 (non-functional) (Fragment)	299.80	9.48
A0A075B7B8	Immunoglobulin heavy variable 3/OR16-12 (non-functional) (Fragment)	242.49	9.40
A0A075B7E8	Immunoglobulin heavy variable 3/OR16-13 (non-functional) (Fragment)	242.49	9.40
S4R460	Immunoglobulin heavy variable 3/OR16-9 (non-functional)	5489.71	31.25
P01762	Immunoglobulin heavy variable 3-11	299.80	9.40
P01766	Immunoglobulin heavy variable 3-13	299.80	9.48
A0A0C4DH32	Immunoglobulin heavy variable 3-20 (Fragment)	299.80	9.40
A0A0B4J1V1	Immunoglobulin heavy variable 3-21	299.80	9.40
P01764	Immunoglobulin heavy variable 3-23	242.49	12.82
P01768	Immunoglobulin heavy variable 3-30	242.49	31.62
P01772	Immunoglobulin heavy variable 3-33	242.49	31.62
A0A0B4J1X8	Immunoglobulin heavy variable 3-43	299.80	9.32
P01763	Immunoglobulin heavy variable 3-48	299.80	9.40
P01767	Immunoglobulin heavy variable 3-53	242.49	12.93
A0A0C4DH42	Immunoglobulin heavy variable 3-66	242.49	12.93
P01780	Immunoglobulin heavy variable 3-7	299.80	9.40
A0A0B4J1X5	Immunoglobulin heavy variable 3-74	242.49	9.40
P01782	Immunoglobulin heavy variable 3-9	299.80	9.32
P01591	Immunoglobulin J chain	20006.96	49.06
P01834	Immunoglobulin kappa constant	28856.88	82.24
A0A0C4DH90	Immunoglobulin kappa variable 3/OR2-268 (non-functional) (Fragment)	362.90	7.76
P04433	Immunoglobulin kappa variable 3-11	1198.54	26.09
P01624	Immunoglobulin kappa variable 3-15	362.90	7.83
A0A075B6H7	Immunoglobulin kappa variable 3-7 (non-functional) (Fragment)	362.90	7.76
A0A0A0MRZ8	Immunoglobulin kappa variable 3D-11	1198.54	26.09
A0A0C4DH55	Immunoglobulin kappa variable 3D-7	362.90	7.56
P06312	Immunoglobulin kappa variable 4-1	250.98	19.83
P0CG04	Immunoglobulin lambda constant 1	40610.55	77.36
P0DOY2	Immunoglobulin lambda constant 2	44714.51	93.40
P0DOY3	Immunoglobulin lambda constant 3	44714.51	93.40
P0CF74	Immunoglobulin lambda constant 6	23147.62	50.94
A0M8Q6	Immunoglobulin lambda constant 7	19435.36	36.79
P01715	Immunoglobulin lambda variable 3-1	344.58	38.26
B9A064	Immunoglobulin lambda-like polypeptide 5	40610.55	38.32
Q9BQU0	Inhibitory NK receptor	242.62	11.21
Q9NVH2	Integrator complex subunit 7	267.39	4.26
Q01638	Interleukin-1 receptor-like 1	304.24	7.01
H0YNL8	Iron-responsive element-binding protein 2	377.91	29.09
A0A0G2JPA6	Killer cell immunoglobulin-like receptor 3DL2	242.62	11.64
P22079	Lactoperoxidase	2259.91	35.11
P02788	Lactotransferrin	862.74	28.59
A6NMS7	Leucine-rich repeat-containing protein 37A	263.12	1.71
A6NM11	Leucine-rich repeat-containing protein 37A2	252.18	1.71
O60309	Leucine-rich repeat-containing protein 37A3	276.06	4.53
P31025	Lipocalin-1	14925.97	53.98
Q86W92	Liprin-beta-1	292.75	10.29
P00338	L-lactate dehydrogenase A chain	196.57	21.69
Q9BY66	Lysine-specific demethylase 5D	307.10	8.58
P61626	Lysozyme C	15283.53	66.89
P14174	Macrophage migration inhibitory factor	616.56	47.83
C9JF79	Malate dehydrogenase (Fragment)	263.72	11.71
P40925	Malate dehydrogenase_ cytoplasmic	653.55	11.38
Q5HYA8	Meckelin	241.84	1.61
Q9Y4B5	Microtubule cross-linking factor 1	26.23	1.52
Q8TAX7	Mucin-7	13700.40	9.28
U3KPS2	Myeloblastin	554.69	17.67
P24158	Myeloblastin	631.43	28.52
Q9NYA4	Myotubularin-related protein 4	315.44	7.11
P59665	Neutrophil defensin 1	1789.52	25.53
P59666	Neutrophil defensin 3	1789.52	25.53
Q9BYH8	NF-kappa-B inhibitor zeta	371.15	4.32
Q2L696	Nucb2 splice variant	663.95	25.13
A0A087WSV8	Nucleobindin 2_ isoform CRA_b	954.67	25.48
P80303	Nucleobindin-2	954.67	25.48
P04746	Pancreatic alpha-amylase	88276.59	55.97
P42338	Phosphatidylinositol 4_5-bisphosphate 3-kinase catalytic subunit beta isoform	561.79	6.26
A0A0A0MRF9	Phosphoinositide phospholipase C	313.90	4.55
P13796	Plastin-2	283.93	25.04
Q86YL7	Podoplanin	866.94	34.57
P11940	Polyadenylate-binding protein 1	582.59	10.69
E7ERJ7	Polyadenylate-binding protein	582.59	11.26
Q8NDX5	Polyhomeotic-like protein 3	348.42	3.05
P01833	Polymeric immunoglobulin receptor	12791.93	57.98
Q8TCS8	Polyribonucleotide nucleotidyltransferase 1_ mitochondrial	32.49	3.19
Q6S8J3	POTE ankyrin domain family member E	4118.47	13.86
A5A3E0	POTE ankyrin domain family member F	4040.70	11.72
P0CG38	POTE ankyrin domain family member I	3413.22	4.74
P0CG39	POTE ankyrin domain family member J	2796.68	3.85
Q8TED1	Probable glutathione peroxidase 8	341.42	17.22
Q8IZM9	Probable sodium-coupled neutral amino acid transporter 6	426.92	6.80
K7EJ44	Profilin	470.78	37.50
P07737	Profilin-1	910.82	49.29
P12273	Prolactin-inducible protein	30448.27	76.71
A0A0A0MT31	Proline-rich protein 4	23475.68	72.29
P07602	Prosaposin	510.46	39.12
Q5W0V3	Protein FAM160B1	862.81	23.66
Q6P5S2	Protein LEG1 homolog	6592.09	36.97
Q9H7Z3	Protein NRDE2 homolog	45.79	1.20
P05109	Protein S100-A8	3184.30	23.66
P06702	Protein S100-A9	1737.55	77.19
O14795	Protein unc-13 homolog B	59.05	1.19
H3BQ60	Puratrophin-1 (Fragment)	266.79	50.00
Q9BYX7	Putative beta-actin-like protein 3	2063.16	10.67
Q5VSP4	Putative lipocalin 1-like protein 1	3097.31	11.11
P52566	Rho GDP-dissociation inhibitor 2	1026.87	30.35
P35913	Rod cGMP-specific 3'_5'-cyclic phosphodiesterase subunit beta	374.14	8.08
P02810	Salivary acidic proline-rich phosphoprotein 1/2	4566.91	72.29
P02787	Serotransferrin	4566.92	48.42
P02768	Serum albumin	63281.61	75.04
O00193	Small acidic protein	258.69	13.11
P02808	Statherin	41653.6	48.39
P02814	Submaxillary gland androgen-regulated protein 3B	20898.6	65.82
Q9UH99	SUN domain-containing protein 2	70.82	1.67
A0A075B6V5	T cell receptor alpha variable 36/delta variable 7 (Fragment)	278.89	24.78
Q7Z6L1	Tectonin beta-propeller repeat-containing protein 1	384.23	7.12
F2Z350	Testis-expressed protein 29	447.37	32.14
Q7Z4L5	Tetratricopeptide repeat protein 21B	78.57	4.56
P20061	Transcobalamin-1	378.51	22.86
P29401	Transketolase	676.10	30.98
Q6ZMR5	Transmembrane protease serine 11A	281.15	11.16
P02766	Transthyretin	438.46	44.22
P60174	Triosephosphate isomerase	651.56	36.36
O43818	U3 small nucleolar RNA-interacting protein 2	297.84	16.00
A0A0J9YY99	Uncharacterized protein (Fragment)	242.49	12.82
H7C2Y3	Uncharacterized protein C2orf80 (Fragment)	318.87	16.41
H0Y8H3	Uncharacterized protein C3orf67 (Fragment)	590.54	74.68
A0A087WZY1	Uncharacterized protein	22581.8	16.60
A0A0G2JMZ2	Uncharacterized protein	252.18	1.71
A0A0G2JRT3	Uncharacterized protein	252.18	1.77
P02774	Vitamin D-binding protein	245.21	21.52
Q14508	WAP four-disulfide core domain protein 2	935.99	33.87
Q9UDV6	Zinc finger protein 212	424.39	16.97
P25311	Zinc-alpha-2-glycoprotein	2292.60	31.54
Q96DA0	Zymogen granule protein 16 homolog B	46355.09	58.17

**Table 4:** Proteins of the saliva identified in only in the individual analysis

Accession number	Protein name	score	Cover(%)
P31947	14-3-3 protein sigma	297.17	24.60
O00231	26S proteasome non-ATPase regulatory subunit 11	453.07	10.66
P68032	Actin_ alpha cardiac muscle 1	7799.84	26.53
P68133	Actin_ alpha skeletal muscle	7799.84	26.53
P62736	Actin_ aortic smooth muscle	7555.95	23.61
P60709	Actin_ cytoplasmic 1	17763.84	65.60
P63261	Actin_ cytoplasmic 2	17763.84	65.60
P63267	Actin_ gamma-enteric smooth muscle	7555.95	23.67
Q0VD77	ADAMTS-like protein 5	410.00	32.06
P00813	Adenosine deaminase	350.67	12.67
O60503	Adenylate cyclase type 9	471.53	5.69
Q99996	A-kinase anchor protein 9	34.16	3.58
C9JKR2	Albumin_ isoform CRA_k	29220.48	74.82
P01009	Alpha-1-antitrypsin	413.67	11.24
P01023	Alpha-2-macroglobulin	445.71	15.33
A8K2U0	Alpha-2-macroglobulin-like protein 1	148.51	10.32
P04745	Alpha-amylase 1	97076.24	78.86
P19961	Alpha-amylase 2B	77429.32	62.82
P06733	Alpha-enolase	1439.59	49.08
Q8N6M6	Aminopeptidase O	261.58	10.13
Q01484	Ankyrin-2	39.24	4.22
P02652	Apolipoprotein A-II	941.64	47.00
Q14562	ATP-dependent RNA helicase DHX8	365.21	7.38
Q8IYB8	ATP-dependent RNA helicase SUPV3L1_ mitochondrial	331.22	7.00
P04280	Basic salivary proline-rich protein 1	8867.97	44.39
P02812	Basic salivary proline-rich protein 2	54196.77	69.71
I3L192	Basigin (Fragment)	185.70	16.88
P61769	Beta-2-microglobulin	2754.07	54.62
Q562R1	Beta-actin-like protein 2	1943.05	10.90
P13929	Beta-enolase	131.58	7.60
O95342	Bile salt export pump	495.58	8.18
Q96DR5	BPI fold-containing family A member 2	6426.16	43.37
Q8N4F0	BPI fold-containing family B member 2	6613.00	37.99
Q9NQY0	Bridging integrator 3	398.03	11.46
Q8N4G4	CA6 protein	294.75	4.47
O75808	Calpain-15	215.66	3.68
P23280	Carbonic anhydrase 6	9824.04	57.47
Q0P665	CCDC88C protein	188.41	0.00
Q8N163	Cell cycle and apoptosis regulator protein 2	573.49	11.05
O14647	Chromodomain-helicase-DNA-binding protein 2	250.16	2.84
H0Y7A8	Chromosome 9 open reading frame 3 (Fragment)	236.18	19.31
P35606	Coatomer subunit beta'	189.71	2.21
A2ABG0	Complement C2 (Fragment)	409.38	20.25
P01024	Complement C3	526.68	24.53
Q53SF7	Cordon-bleu protein-like 1	168.78	4.32
P04080	Cystatin-B	1041.42	70.41
P01034	Cystatin-C	3437.76	51.37
P28325	Cystatin-D	2141.16	75.35
P01036	Cystatin-S	28189.63	76.60
P09228	Cystatin-SA	13641.19	67.38
P01037	Cystatin-SN	28293.31	70.21
P54108	Cysteine-rich secretory protein 3	373.11	34.29
Q8NF50	Dedicator of cytokinesis protein 8	351.74	5.72
Q9UGM3	Deleted in malignant brain tumors 1 protein	285.95	7.05
Q5TBH6	Dihydroxyacetone phosphate acyltransferase (Fragment)	182.78	23.42
P28340	DNA polymerase delta catalytic subunit	269.07	5.15
M0R2B7	DNA polymerase	269.07	5.03
Q5T4S7	E3 ubiquitin-protein ligase UBR4	22.83	2.70
Q92838	Ectodysplasin-A	258.64	15.86
Q8N3D4	EH domain-binding protein 1-like protein 1	260.45	4.33
Q6P179	Endoplasmic reticulum aminopeptidase 2	522.88	7.92
Q7L775	EPM2A-interacting protein 1	277.97	2.80
Q9H501	ESF1 homolog	205.30	12.22
A0A1B0GUN9	Espin	59.79	6.02
Q8IXL6	Extracellular serine/threonine protein kinase FAM20C	322.36	5.48
Q01469	Fatty acid-binding protein_ epidermal	444.20	32.59
Q9BZK7	F-box-like/WD repeat-containing protein TBL1XR1	376.57	15.18
P02675	Fibrinogen beta chain	187.44	13.03
P15328	Folate receptor alpha	400.38	35.80
Q8NHY3	GAS2-like protein 2	287.31	6.14
P06396	Gelsolin	427.99	17.77
O14893	Gem-associated protein 2	443.14	31.07
P53611	Geranylgeranyl transferase type-2 subunit beta	470.85	16.92
P06744	Glucose-6-phosphate isomerase	787.26	28.49
P04406	Glyceraldehyde-3-phosphate dehydrogenase	793.86	39.40
O95427	GPI ethanolamine phosphate transferase 1	233.92	7.73
Q8IWJ2	GRIP and coiled-coil domain-containing protein 2	22.31	1.25
P00738	Haptoglobin	1233.11	55.42
P00739	Haptoglobin-related protein	281.28	15.52
G3V1N2	HCG1745306_ isoform CRA_a	15851.36	94.55
E7BWR8	HCG2043595_ isoform CRA_a	252.74	7.76
P69905	Hemoglobin subunit alpha	16443.62	83.80
P68871	Hemoglobin subunit beta	22740.65	95.24
P02042	Hemoglobin subunit delta	5150.58	39.46
P02100	Hemoglobin subunit epsilon	2097.61	6.80
P69891	Hemoglobin subunit gamma-1	2097.61	6.80
P69892	Hemoglobin subunit gamma-2	2097.61	6.80
P15515	Histatin-1	5208.41	36.84
P15516	Histatin-3	4795.66	13.73
E9PRF4	Histone-lysine N-methyltransferase (Fragment)	316.72	3.89
Q15047	Histone-lysine N-methyltransferase SETDB1	316.72	3.80
P47902	Homeobox protein CDX-1	196.38	26.04
P31270	Homeobox protein Hox-A11	264.91	14.38
P09630	Homeobox protein Hox-C6	93.47	4.68
Q4G0P3	Hydrocephalus-inducing protein homolog	264.63	2.46
A0A0G2JMB2	Ig alpha-2 chain C region (Fragment)	48303.27	79.12
A0A0A0MS07	Ig gamma-1 chain C region (Fragment)	3209.86	45.76
A0A087WYJ9	Ig mu chain C region	3019.36	54.87
P04220	Ig mu heavy chain disease protein	2170.36	39.90
P01876	Immunoglobulin heavy constant alpha 1	40927.72	84.42
P01877	Immunoglobulin heavy constant alpha 2	28394.92	68.53
P01857	Immunoglobulin heavy constant gamma 1	5891.82	50.91
P01859	Immunoglobulin heavy constant gamma 2	1360.10	31.29
P01860	Immunoglobulin heavy constant gamma 3	1756.61	30.24
P01861	Immunoglobulin heavy constant gamma 4	1509.92	30.89
P01871	Immunoglobulin heavy constant mu	3019.36	54.75
A0A075B7D0	Immunoglobulin heavy variable 1/OR15-1 (non-functional) (Fragment)	252.28	10.26
A0A075B7F0	Immunoglobulin heavy variable 3/OR16-10 (non-functional) (Fragment)	3426.81	13.79
S4R460	Immunoglobulin heavy variable 3/OR16-9 (non-functional)	8502.51	36.46
P01762	Immunoglobulin heavy variable 3-11	3426.81	23.08
P01766	Immunoglobulin heavy variable 3-13	3426.81	13.79
A0A0C4DH32	Immunoglobulin heavy variable 3-20 (Fragment)	3426.81	13.68
A0A0B4J1V1	Immunoglobulin heavy variable 3-21	3426.81	23.08
A0A0B4J1X8	Immunoglobulin heavy variable 3-43	3426.81	13.56
P01763	Immunoglobulin heavy variable 3-48	3426.81	23.08
P01780	Immunoglobulin heavy variable 3-7	3426.81	23.08
P01782	Immunoglobulin heavy variable 3-9	3426.81	13.56
A0A0B4J1U7	Immunoglobulin heavy variable 6-1	294.24	5.79
P01591	Immunoglobulin J chain	21280.25	68.55
P01834	Immunoglobulin kappa constant	37053.21	85.98
P04433	Immunoglobulin kappa variable 3-11	1303.48	26.09
P01619	Immunoglobulin kappa variable 3-20	868.06	7.76
A0A0A0MRZ8	Immunoglobulin kappa variable 3D-11	1303.48	26.09
P06312	Immunoglobulin kappa variable 4-1	423.92	19.83
P0CG04	Immunoglobulin lambda constant 1	33910.90	77.36
P0DOY2	Immunoglobulin lambda constant 2	40674.07	77.36
P0DOY3	Immunoglobulin lambda constant 3	40674.07	77.36
P0CF74	Immunoglobulin lambda constant 6	30147.40	50.94
A0M8Q6	Immunoglobulin lambda constant 7	22557.57	36.79
B9A064	Immunoglobulin lambda-like polypeptide 5	33910.9	38.32
P06870	Kallikrein-1	196.20	10.31
P43626	Killer cell immunoglobulin-like receptor 2DL1	252.74	7.76
A0A0G2JNJ6	Killer cell immunoglobulin-like receptor 2DS1	325.76	16.62
Q9HAQ2	Kinesin-like protein KIF9	158.59	4.43
B4DZK5	Kinesin-like protein	133.37	10.51
Q6H2H3	KIR2DL1	252.74	7.76
P22079	Lactoperoxidase	1577.03	41.43
P02788	Lactotransferrin	1069.99	35.21
Q6PKG0	La-related protein 1	139.16	6.20
P09960	Leukotriene A-4 hydrolase	225.55	19.31
P31025	Lipocalin-1	8361.36	51.14
P00338	L-lactate dehydrogenase A chain	986.52	20.78
Q9BYZ2	L-lactate dehydrogenase A-like 6B	323.86	8.66
Q9BY66	Lysine-specific demethylase 5D	59.78	0.78
P61626	Lysozyme C	9288.56	54.05
P14174	Macrophage migration inhibitory factor	254.18	55.65
P14780	Matrix metalloproteinase-9	225.62	15.13
Q96JG8	Melanoma-associated antigen D4	150.96	6.07
P01033	Metalloproteinase inhibitor 1	445.25	29.95
Q96GX9	Methylthioribulose-1-phosphate dehydratase	198.99	23.14
O15021	Microtubule-associated serine/threonine-protein kinase 4	168.04	3.43
O43283	Mitogen-activated protein kinase kinase kinase 13	533.35	8.70
Q8TAX7	Mucin-7	10429.01	15.65
Q8NI22	Multiple coagulation factor deficiency protein 2	260.43	23.97
O75970	Multiple PDZ domain protein	43.13	2.32
P24158	Myeloblastin	341.23	17.19
P59665	Neutrophil defensin 1	2353.04	15.96
P59666	Neutrophil defensin 3	2353.04	15.96
P04746	Pancreatic alpha-amylase	64829.77	60.27
Q08752	Peptidyl-prolyl cis-trans isomerase D	470.08	17.57
P13796	Plastin-2	531.41	28.87
P01833	Polymeric immunoglobulin receptor	16305.42	45.42
Q6S8J3	POTE ankyrin domain family member E	3659.07	9.49
A5A3E0	POTE ankyrin domain family member F	3575.10	10.14
P0CG38	POTE ankyrin domain family member I	2591.40	5.67
P0CG39	POTE ankyrin domain family member J	1362.79	4.82
P17844	Probable ATP-dependent RNA helicase DDX5	220.36	4.89
I3L3D5	Profilin (Fragment)	1209.81	10.91
P07737	Profilin-1	1209.81	20.71
P12273	Prolactin-inducible protein	22984.41	89.04
A0A0A0MT31	Proline-rich protein 4	52615.69	72.29
P07602	Prosaposin	316.92	22.52
Q9P219	Protein Daple	206.07	0.69
P49354	Protein farnesyltransferase/geranylgeranyltransferase type-1 subunit alpha	1184.15	17.41
Q6P5S2	Protein LEG1 homolog	7928.19	40.00
Q9H7Z3	Protein NRDE2 homolog	339.41	6.79
Q8WYL5	Protein phosphatase Slingshot homolog 1	286.92	2.38
O43663	Protein regulator of cytokinesis 1	83.55	7.42
P05109	Protein S100-A8	1391.46	31.18
P06702	Protein S100-A9	2043.00	78.07
Q9NQW1	Protein transport protein Sec31B	442.02	7.63
Q92954	Proteoglycan 4	188.50	2.78
Q96MK3	Pseudokinase FAM20A	287.95	8.50
Q9BYX7	Putative beta-actin-like protein 3	1353.87	29.07
Q5VSP4	Putative lipocalin 1-like protein 1	3095.80	11.11
Q5JXB2	Putative ubiquitin-conjugating enzyme E2 N-like	341.70	32.03
A4QN01	Putative uncharacterized protein encoded by LINC01553	191.02	19.53
Q15276	Rab GTPase-binding effector protein 1	211.79	8.58
Q9Y2J0	Rabphilin-3A	47.85	7.93
Q14699	Raftlin	796.05	17.30
G3XAJ6	Raft-linking protein_ isoform CRA_c	779.81	13.84
P52565	Rho GDP-dissociation inhibitor 1	251.72	19.61
Q8IXT5	RNA-binding protein 12B	263.79	6.39
K4DI92	RWD domain containing 4A	636.75	30.48
Q6NW29	RWD domain-containing protein 4	636.75	30.32
P02810	Salivary acidic proline-rich phosphoprotein 1/2	52615.69	72.29
Q9BZL6	Serine/threonine-protein kinase D2	403.28	9.68
B4DTS2	Serine/threonine-protein kinase	401.26	9.57
P02787	Serotransferrin	4390.41	39.26
P02768	Serum albumin	64055.35	79.80
P02808	Statherin	25654.54	48.39
P02814	Submaxillary gland androgen-regulated protein 3B	50678.11	65.82
P00441	Superoxide dismutase [Cu-Zn]	1005.47	45.45
H0YN01	Talin-2	197.30	34.55
Q92609	TBC1 domain family member 5	344.39	5.16
Q7Z6L1	Tectonin beta-propeller repeat-containing protein 1	62.51	2.49
Q6N022	Teneurin-4	64.41	4.15
P10599	Thioredoxin	300.36	32.38
Q96J01	THO complex subunit 3	335.46	20.51
Q5JTD0	Tight junction-associated protein 1	432.54	3.95
P37837	Transaldolase	676.70	23.74
P20061	Transcobalamin-1	670.49	33.26
A6H8Y1	Transcription factor TFIIIB component B'' homolog	67.01	6.17
P29401	Transketolase	1109.18	29.05
Q9C0B7	Transport and Golgi organization protein 6 homolog	101.09	8.78
P60174	Triosephosphate isomerase	582.07	15.73
P07437	Tubulin beta chain	251.86	5.86
Q13885	Tubulin beta-2A chain	268.91	5.84
Q9BVA1	Tubulin beta-2B chain	251.86	5.84
P04350	Tubulin beta-4A chain	242.62	5.86
P68371	Tubulin beta-4B chain	242.62	5.84
H3BLT7	Tubulin monoglycylase TTLL3 (Fragment)	205.55	1.15
Q9NVE5	Ubiquitin carboxyl-terminal hydrolase 40	49.55	6.15
Q70EL2	Ubiquitin carboxyl-terminal hydrolase 45	709.84	12.04
D6RC01	Ubiquitinyl hydrolase 1	685.20	10.14
B4DSH7	UDP-galactose translocator	296.27	22.16
H7C2Y3	Uncharacterized protein C2orf80 (Fragment)	203.05	50.78
Q9H1L0	Uncharacterized protein MIR1-1HG	440.61	32.48
A0A087WZY1	Uncharacterized protein	50162.86	16.60
J3QRI8	UPF0183 protein C16orf70 (Fragment)	350.13	32.65
Q13488	V-type proton ATPase 116 kDa subunit a isoform 3	105.99	9.40
Q14508	WAP four-disulfide core domain protein 2	2122.26	33.87
Q9NXC5	WD repeat-containing protein mio	208.07	1.94
Q9BUG6	Zinc finger and SCAN domain-containing protein 5A	97.41	13.71
Q8N8U3	Zinc finger CCHC domain-containing protein 5	189.02	7.79
Q9H0M4	Zinc finger CW-type PWWP domain protein 1	242.57	7.10
Q9NWS9	Zinc finger protein 446	77.75	7.56
P25311	Zinc-alpha-2-glycoprotein	1420.80	28.19
Q96DA0	Zymogen granule protein 16 homolog B	32673.11	56.73

## Discussion

This study aimed at standardizing a protocol for proteomic analysis of saliva that is sensitive, easy to perform and of low cost, to be used in future experiments involving quantitative shotgun proteomics. The first issue to be solved was related to the necessity of depletion of highly abundant proteins in saliva, such as albumin and IgG^8^ ^,^ ^14^ that could mask and make difficult the identification of low abundance biomarkers. Krief and collaborators^7^ (2011) evaluated whether depletion of salivary amylase, albumin and IgGs could improve the ability to visualize proteins in two-dimensional gel electrophoresis (2-DE) in oral fluids. They observed 36 new spots after depletion, and 58 spots showed more than twofold increase intensity after depletion^7^. Therefore, we hypothesized that this better identification profile could occur not only in two-dimensional gel electrophoresis (2-DE), but also in shotgun proteomics, when albumin and IgG were depleted. Thus, in the first test, we compared the use or not of the albumin and IgG depletion column after the extraction process of the salivary proteins. For this, we used a pool of ten saliva samples. When the column was used, only 35 proteins were identified (Table 1). This figure increased to 248 when the column was not used (Table 2). We believe this occurred because, when using the albumin and IgG depletion column, there was also depletion of other proteins, since using the column increases one more process in the methodology. We also believe that many proteins could bind to albumin and IgGs, thus being depleted together. Among the identified proteins, in both situations, are those typically found in saliva. By contrast, when the depletion column was used, classical salivary proteins such as 14-3-3 proteins, histatins, statherin, lactoperoxidase, lactotransferrin, lysozyme C, neutrophil defensins, protein S100A9, serotransferrin and some cystatin isoforms were not identified. Thus, contrary to what was observed in gel-based proteomics^7^, in shotgun proteomics the use of albumin and IgG depletion column impaired protein identification according to our workflow. Some studies, in spite of that, report advantages in using depletion columns when more than one workflow is employed^14^. However, this increases the time and cost of the analysis.

In the second test, we compared analysis of pooled samples (from ten individuals) *versus* individual analysis, without using the depletion column. In the individual analysis 239 proteins were identified (Table 4), while 212 proteins were identified in the pooled sample (Table 3). One-hundred and twenty three proteins were common to both groups (data not shown), and among them are most of the proteins typically found in saliva. The proteins exclusively found in the individual sample or in the pooled sample are not typically reported in saliva, which might be related to individual variation. The analysis of individual samples is important to allow confident comparison among the groups under study, especially in quantitative shotgun proteomics.

Generally, the methodologies used in proteomics are classified into two main categories: the bottom-up, which is also called shotgun proteomics, or top-down proteomics. Both methodologies have advantages and limitations, and their employment depends on the treatment given to the sample^9^. Shotgun proteomics is characterized by analyzing samples after proteolytic digestion in peptides, which is typically performed with trypsin^2^ ^,^ ^9^, while the top-down proteome of a sample involves analysis of intact proteins^9^. In shotgun proteomics, proteins from a complex mixture are digested, and the resulting peptides are analyzed by mass spectrometry. One of the advantages of this strategy is to investigate a large number of proteins regardless of their size. The limitations are related to incomplete coverage of the protein sequence, loss of post-translational modifications and degradation because of proteolytic digestion^4^ ^,^ ^9^. The top-down proteomics differs from the shotgun as it explores intact proteins by injecting the proteins into the mass spectrometer without performing digestion, minimizing any change in the sample and allowing a better characterization of post-translational modifications, especially those related to naturally occurring cleavages and alternative splicing^3^, avoiding interference problems based on peptides and allowing deducing the primary structure of the protein^4^ ^,^ ^9^. However, this technique is considered bounded by the collision energy required in protein fragmentation, which is insufficient for proteins greater than 50 KDa, and its application is restricted to the analysis of purified proteins^4^ ^,^ ^9^ ^,^ ^11^. In addition, top-down proteomics method requires the use of one or more forms of separation prior to mass spectrometry analysis^12^. Moreover, top-down platforms are intrinsically limited by the sample treatments required for use in mass spectrometry, involving the use of acids such as formic and trifluoroacetic acid^9^ ^,^ ^12^ ^,^ ^19^, which inevitably exclude proteins that are insoluble in acidic solutions. In addition, intact high molecular weight proteins and heterogeneous glycosylated proteins are not accessible in their naturally occurring form, even to the best level of mass spectrometry^2^.

Previous studies demonstrated that top-down platforms cannot achieve the same coverage of shotgun platforms for different reasons, such as: i) the intact protein must be soluble in the acid solution compatible with an ESI-MS analysis; (ii) the protein should not be heterogeneous (glycosylated isoforms), because in this case the intact protein mass cannot be deduced by the ESI spectrum; (iii) protein dimensions have to be limited, because MS-MS fragmentation spectra are too complex to be interpreted^3^ ^,^ ^15^. Nonetheless, the top-down strategy may reveal the richness of the isoform and the diversity of post-translational modifications, which in the shotgun proteomics strategy may result in the relevant loss of this molecular information^2^ ^,^ ^3^. Thus, shotgun proteomics may exhibit this deficiency in the human saliva proteome, in which many proteins such as basic PRPs and acids are not very susceptible to the proteolytic enzymes action and reveal very similar sequences. Therefore, many fragments cannot be related to a specific original protein. However, the shotgun platforms showed the best performance in terms of number of components detected, because the sensitivity of mass spectrometry is sufficient to reveal thousands of peptides in a single analysis. In this way, shotgun proteomics covers the largest variety of detectable components, regardless of their mass, due to the proteolytic digestion of large proteins almost always generates peptides that can disclose the presence of the protein in a complex mixture. Due to these reasons, the number of salivary components currently detectable by shotgun proteomics approaches is more than five times greater than that of components detected by any other platform^2^ ^,^ ^10^. Thus, in this study we employed shotgun proteomics.

Based on the results of the two tests, the protocol for salivary shotgun proteomic analysis was satisfactory, since it allowed the identification of many proteins, including those typically found in saliva. Moreover, it is easy to perform and cheaper than the methods previously described, since it does not require the use of depletion columns. Furthermore, it allows individual analysis of the samples, which is very important in quantitative proteomics. Thus, this protocol could be used in future studies involving shotgun proteomic analysis of saliva.
